# Patterns of brain activity in choice or instructed go and no-go tasks

**DOI:** 10.1007/s00221-025-07027-6

**Published:** 2025-02-21

**Authors:** Sanaz Attaripour Isfahani, Patrick McGurrin, Felipe Vial, Mark Hallett

**Affiliations:** 1 Human Motor Control Section, National Institutes of Health, National Insitute of Neurological Disorders and Stroke, Building 10, Room 7D37, 10 Center Drive, Bethesda, MD 20892-1428 USA; 2 Department of Neurology, University of California, Irvine. 200 S. Manchester Ave., Ste 206, Orange, CA 92868 USA; 3 Facultad de Medicina Clínica Alemana, Universidad del Desarrollo, 5951, Av Vitacura, Vitacura, Región Metropolitana Chile

**Keywords:** Contingent negative variation, Go/no-go task, Reaction time, Decision, Free will, Motor control

## Abstract

The goal of this study was to investigate the decision making process for choosing what movements to make. We used electroencephalography (EEG) to investigate patterns of the contingent negative variation (CNV) associated with free-choice decisions to move or abstain, comparing them to conditions where actions were commanded. Our primary hypothesis was that choice tasks would differ significantly from each other and exhibit EEG patterns akin to their command-driven counterparts after the decisions were made, at least, in the 50 ms block of time prior to movement. A secondary analysis evaluated post hoc comparisons of time, in 50 ms blocks, to understand the temporal development of the CNV for each condition. We also conducted an exploratory analysis of EEG event-related desynchronization (ERD) to identify patterns of brain activity associated with the decision-making process. This approach was taken due to the exploratory nature of our hypotheses concerning the spatial and temporal characteristics of EEG activity during these free-choice versus commanded tasks. We studied 12 right-handed healthy volunteers (7 women, mean age 53 years, range 39–73 years) with no prior history of neurological or major psychiatric illness. A CNV paradigm encompassing commanded and choice tasks was devised, with a 2500 ms interval between S1 and S2, while recording EEG and electromyography (EMG). S1 provided full information about the upcoming task, which was to be executed at the time of S2. We assessed CNV and explored whole scalp EEG activity, including both voltage as well as power in the alpha and beta frequency ranges. Clear and similar CNVs were observed for command and choice go tasks prior to the movements, contrasting with near-zero CNVs for the command and choice no-go tasks. Separation of CNVs for command go and no-go tasks occurred around 1600 ms post-S1, and choice CNVs separated about 2150 ms post-S1. Exploratory analysis revealed that beta power provided information about decision and preparation processes much earlier. The left dorsolateral prefrontal cortex (DLPFC) exhibited the initial sign of decision approximately 500 ms post-S1 for all tasks, with subsequent preparation for movement or restraint involving distinct activity in various brain regions. The localization of effects in the left DLPFC was determined by visual analysis of the informative electrode sites. The CNVs separate about 2 s after S1, and it appears that this process represents preparation for movement (or no movement). Exploration of the beta activity suggests an earlier decision process which leads eventually to subsequent task preparation and activation. Choice decisions lag slightly behind command decisions, with the CNV apparently reflecting motor implementation rather than the decision-making process. In a simple motor task with an exploratory analysis, both commanded and choice-based decisions are rapidly initiated in the left DLPFC. While the CNV distinguishes between go and no-go conditions, it primarily appears to signify preparation for implementation of the task following the earlier decision. Further controlled studies will be needed to confirm these results.

**Supplementary Information:**

The online version contains supplementary material available at 10.1007/s00221-025-07027-6.

## Introduction

Making decisions is an important function of the brain. Moreover, the ability to make decisions is a factor that gives people the sense of free will. They can choose to do something when there is also a choice to do otherwise. How the brain makes decisions has been intensively studied, but the process is not yet fully understood.

The experimental paradigm of the contingent negative variation (CNV) gives the opportunity to explore the sequential brain processes during the decision-making process for making movements. The paradigm has a first stimulus (S1) and a second stimulus (S2). S1 can serve as a simple warning or have either partial or full information about what movement is to be made. S2 is the imperative stimulus for the movement and may contain additional information if necessary to fully describe the desired movement. The CNV is a slow negative electroencephalographic (EEG) potential elicited between the S1 and S2 stimuli when a motor response to S2 is required (Rohricht et al. 2018). Initiating and canceling an action, typically considered distinct processes, may actually be closely related. (Du et al. 2024). The CNV does not only appear when a motor response is required; it is an anticipatory activity that occurs with unpredictable S2 presentations and neutral cues. Often, the CNV has been studied with movement (or no movement) not fully specified until the time of S2. However, one area that has been less explored is that the paradigm also allows giving a choice at S1 that can be acted on at S2, and this would permit the study of the decision process between S1 and S2. It has been demonstrated that the CNV and corresponding power changes, such as those in the alpha and beta bands, can reflect distinct aspects of the underlying neural processes and may not always occur simultaneously during task performance.

The CNV's characteristics depend on task-specific conditions and cognitive contexts, suggesting it reflects a core preparatory process for brain optimization and other cognitive functions. The study challenges the linear relationship between CNV amplitude and behavioral performance, proposing instead that CNV amplitude may vary with different cognitive tasks and conditions. This illustrates the complexity of CNV as a neurophysiological phenomenon. (Kononowicz et al. 2016).

We designed a novel CNV paradigm that included cues to command action or command inaction at S1, and an additional condition in which a free-choice decision of whether or not to move was also presented. This allowed us to compare the processes in a decision to go or not to go with a command to go or not go. We chose a no-go task to compare with a go task, rather than two different go tasks, in order to maximize the difference in motor preparation. No-go is not a passive task; it is active inhibition as demonstrated in many studies (Filipovic et al. 2001; Leocani et al. 2001).

We hypothesized that choosing to go or not go would have unique neural features from one another in the period preceding the S2 cue that would illuminate the decision-making process. We expected that the choice tasks would show similar CNV patterns to that of the corresponding command conditions once the choice had been made. In a second analysis, we also looked at the CNV in 50 ms blocks backward from the time of S2 to see when the CNV might begin to reflect unique features for the command and/or choice tasks. In addition to studying the CNV, we have examined corresponding changes in alpha and beta power, as it has been previously demonstrated that the CNV and these power changes can exhibit different temporal profiles, which may reflect distinct aspects of the underlying neural processes during task performance (Filipovic et al. 2001). This decoupling would suggest that the CNV and event-related desynchronization (ERD) may not always occur simultaneously, indicating that they may represent different neural activities within the same task context. We also performed an exploratory analysis of EEG activity over the whole scalp to evaluate any additional features that may have been present during the decision-making process.

## Methods

### Participants

We studied 12 (7 women) healthy volunteers. All participants were right-handed, as noted during the participant recruitment process. This ensured consistency in the observed neural activity patterns related to motor planning and execution. The mean age of the participants was 53 years (range 39–73 years, SD: 11). They had no prior history of neurological or major psychiatric illness and were not on any CNS active medications. The participants were re-enrolled from the healthy control arm of a study on functional movement disorders at the National Institutes of Health. As part of the baseline screening, they were evaluated by participant- and clinician-rated questionnaires, structured clinical interview (SCID), and structural brain Magnetic Resonance Imaging (MRI), and were deemed to be adequately healthy. They were interviewed and clinically examined again by a neurologist before re-enrollment. All participants gave written informed consent for this study, and the Institutional Review Board at the National Institutes of Health approved all the procedures. Participants were instructed to avoid caffeine and other CNS active substances on the day of the experiment.

### Task and trial presentation

Participants were seated in an armchair in a quiet room with dimmed lights, with their right hand placed in supination with the fingers extended. They were asked to keep their eyes open and fixated on the center of a computer monitor in front of them. The monitor was adjusted to be at a comfortable viewing height for each participant.

**Fig. 1:**
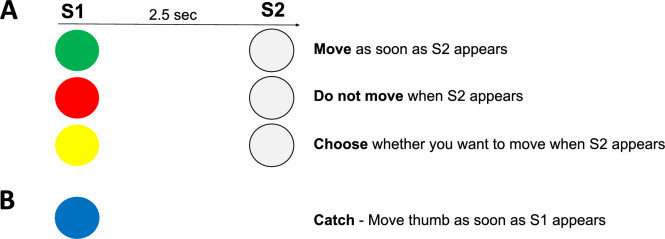
Schematic of the GO No-GO Choice Task. **A** Each trial consisted of the presentation of two stimuli (S1 and S2), which were displayed as colored circles on a monitor placed in front of the participant. Trials were randomized such that the color of the S1 circle could be green, red, or yellow. The green light indicated that the participant should prepare to execute a response, while the red light indicated that neither preparation nor response was required. The yellow light indicated that the participant should choose whether or not to prepare a response for that given trial. Participants were instructed to make their choice at the onset of S1 and express their choice at the onset of S2. S2 was always a white circle and appeared 2.5 s after the onset of S1. Participants were asked to respond (if required given the circle's color) at the onset of S2. The response, when required, was a brisk flexion of the right thumb. **B** Catch trials were also included and were presented as blue circles. During these trials, participants were instructed to execute a brisk flexion of the right thumb as quickly as possible, without waiting for S2. This was designed to ensure sustained attention throughout the experiment

During the experiment, an S1-S2 paradigm with a go/no-go command or go/no-go decision was used for S1 and the cue to implement the commanded or chosen action was S2 (See Fig. 1). S1 and S2 were presented as colored circles (Presentation software, Neurobehavioral Systems) and displayed on a monitor placed in front of the participant. The order of the stimuli and tasks was randomized to ensure counterbalancing between participants.

Trials were randomized such that the color of the S1 circle could be red, green, or yellow (Fig. 1A). The green light indicated that the participant should prepare to execute a response (command go) and a red light indicated that no movement should be made (command no-go). A yellow light indicated that the participant should choose whether to prepare (choice go) or not prepare (choice no-go) a response for that given trial. S2 was always a white circle and was delivered 2.5 s after the onset of S1. Participants were asked to respond, if required given the circle’s color, at the onset of the S2 with a brisk flexion of the right thumb. For the choice trials, there was no instruction about how many trials should be go or no-go, just that both were allowed.

Catch trials were also included and were presented as blue circles (Fig. 1B). Here the participant was instructed to execute a brisk flexion of the right thumb as fast as possible, without waiting for S2. This was to ensure sustained attention throughout the experiment.

Participants performed 5 experimental blocks, each of which consisted of 45 trials. These trials included 20 yellow (choice), 10 green (command go), and 10 red (command no-go) S1 cues. Five catch trials showing a blue circle were also included in each block. The inter-trial interval (measured from S1 onset) randomly varied from 4 to 7 s. Before the start of the experiment, participants performed a practice block after the explanation of the cues and prior to the 5 experimental blocks to ensure optimal task performance during the experiment.

### Recordings

Electromyography (EMG) and Electroencephalography (EEG) were used to record muscle and brain activity during the task, respectively. EEG and EMG were recorded using BrainVision recorder (BrainVision, Morrisville, NC) at a sampling rate of 1 kHz. EEG was recorded using a 64-channel Acticap system, with ground placed at FPz and a left mastoid reference. Impedances were kept below 10 kΩ. EMG data were recorded over the abductor pollicis brevis (APB) muscle using neonatal surface electrodes (3 M, Cardinal Health) and a bipolar montage.

Continuous EEG data were processed using BrainVision Analyzer, EEGLAB (Oostenveld et al. 2011), and custom Matlab scripts (Mathworks, MA). Data were first band-pass filtered (0.1–100 Hz), and artifacts related to eye movements and blinks were subsequently removed using Independent Components Analysis (ICA) (Makeig et al. 1996). Data were epoched around the trial cues, from 1 s prior to S1, to 1.5 s after S2 (total time: 5 s) to isolate the timing of the CNV signal, as well as any pre- or post-cue activity.

After epoching the data, visual trial rejection was performed to remove (1) trials with noisy EEG signals; (2) trials where EMG activity was evident prior to S1; (3) any trials, irrespective of S1 color, where EMG activity was evident between S1 and S2; (4) command no-go trials where EMG activity was evident after S2, and (5) command go trials where EMG activity was not evident. A mean baseline correction was applied (1–50 ms relative to S1) on a trial-by-trial basis and prior to averaging. After these steps, individual trials were averaged by condition for each participant.

### Analysis

Reaction time (RT) was calculated manually for all trials where a response was performed. RT was defined as the time after S2 (S1 in the case of catch trials) where a clear EMG response from the APB muscle was observable from the rectified trace. After collecting the initial data, values greater than 3 standard deviations from the mean were removed during the initial data cleaning process, prior to conducting the main statistical analyses. Following data cleaning, Morlet Wavelet convolution was performed for the frequency analysis of all electrodes, using 40 linearly spaced wavelets between 3 and 30 Hz with several cycles between 4 and 12 (linearly spaced). After convolution, the data were normalized to a baseline window spanning from 1 to 50 ms relative to S1 using division, ensuring consistency across trials. This method was then employed to convert EEG power to event-related desynchronization or synchronization (ERD/ERS) (Pfurtscheller et al. 1999), highlighting task-related activity during each trial relative to the pre-S1 state.

### Statistical analysis

We selected Cz as the single electrode for analysis because it is the predominant electrode used for CNV studies. This provided a clean comparison of the decision portion of the task to previous studies. Pairwise testing was used to compare amplitude values from electrode Cz, using the Tukey HSD test. Data were averaged using an a priori time point of interest, namely over the mean of the last 50 ms prior to S2 for each condition. In addition, post hoc analyses of time–frequency data were also performed using the same period selected for the original analysis. We then performed post hoc comparisons for 50 ms blocks of time moving backward from S2 to understand the temporal development of EEG activity for each condition. For this analysis, an uncorrected p-value of 0.05 was used to indicate statistical significance. For all analyses, values that were greater than 3 standard deviations from the mean were removed.

We conducted an exploratory analysis with EEG and event-related desynchronization (ERD) in the alpha (7–12 Hz) and beta (18–24 Hz) frequencies to identify patterns of brain activity associated with the decision-making process. This approach was taken due to the non-specific nature of our hypotheses concerning the timing (temporal aspect relative to S1, S2) and spatial distribution (electrodes) of EEG changes. The localization of effects in the left DLPFC was determined by analyzing the informative electrode sites identified through anatomical mapping (See Fig. S5) (Cohen 2014; Koessler et al. 2009).

## Results

### Data removal

On average 4.52% (± 0.02) of the trials were removed. The removals were mainly due to noise in the EMG signal, which made recognizing movement onset difficult. Less commonly, removals were because of erroneous responses by the participants, i.e., moving during a command no-go trial, moving before S2, and- less frequently- not moving during a command go trial. Reaction times less than 100 ms (premature responses to S2) and RTs greater than 800 ms (attributed to lapses in attention) were omitted. The low percentage of removed trials was an indication that overall task performance was satisfactory.

### Deciding whether to go or not to go

When considering the choice tasks, participants showed a tendency to choose to move, doing so in 61% of trials. Overall, there were significantly more choice go trials relative to choice-no-go trials (t = 2.482; p = 0.030; Figure S1).

### Reaction time

Mean RT was not significantly different (t = − 0.078; p = 0.449) between command go (222 ± 79 ms) and choice go trials (229 ± 83 ms). However, both of these conditions had RTs that were significantly shorter (both comparisons, p < 0.0001) relative to those for blue catch trials (607 ± 148 ms; Figure S2).

### CNV prior to S2

**Fig. 2:**
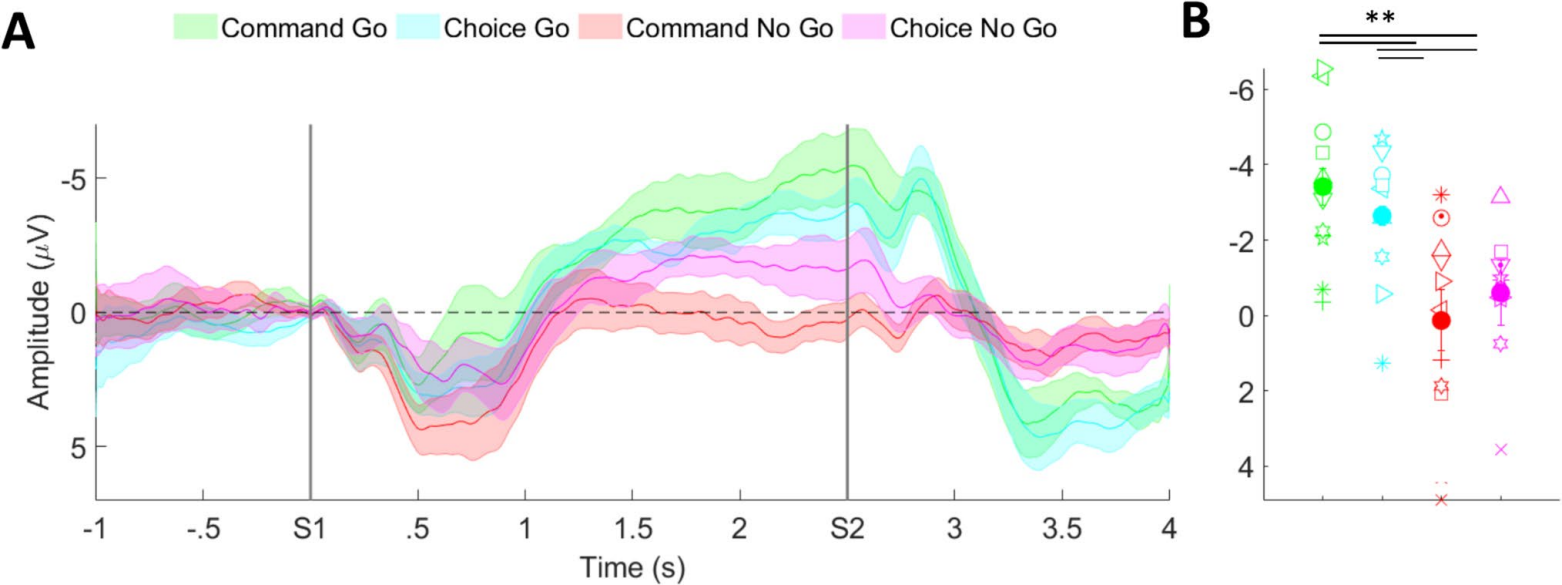
**A** Mean (plus-minus standard error) amplitude data for electrode Cz, normalized to S1, for all participants across all conditions. **B** Average values for a 50 ms period immediately prior to S2. ** indicates statistical significance

We found a significantly higher amplitude for command go than command no-go, in the mean EEG activity over electrode Cz in the 50 ms prior to S2 (t = − 3.657; p = 0.005). We then tested our a priori hypothesis that the amplitude of the CNV in the 50 ms prior to S2 would be significantly different for the choice tasks. There was a significant difference between the choice go and choice no-go tasks (t = − 2.702; p = 0.024). We also compared data within the go and no-go tasks. This revealed no significant difference between the command go and choice go (t = − 2.106; p = 0.172) or between the command no-go and choice no-go (t = − 1.605; p = 0.398) conditions (Fig. 2).

While Cz was the primary outcome, we explored the voltage distribution over the whole scalp (Figure S4). For the go tasks the activity was at Cz from 1500 ms to 2000–2250 ms, but then moved slightly lateral toward C3. For the no-go tasks, no major Cz activity was present at any time, but the voltage appears to be maximal slightly anterior at FCz and Fz. An analysis of CNV over electrode C3 revealed significant differences between the command go and command no-go (t = − 5.07; p < 0.001), and between the choice go and choice no-go (t = − 1.605; p = 0.0398) conditions in the 50 ms period prior to S2 (Figure S3).

#### Temporal differences in CNV prior to S2

**Fig. 3:**
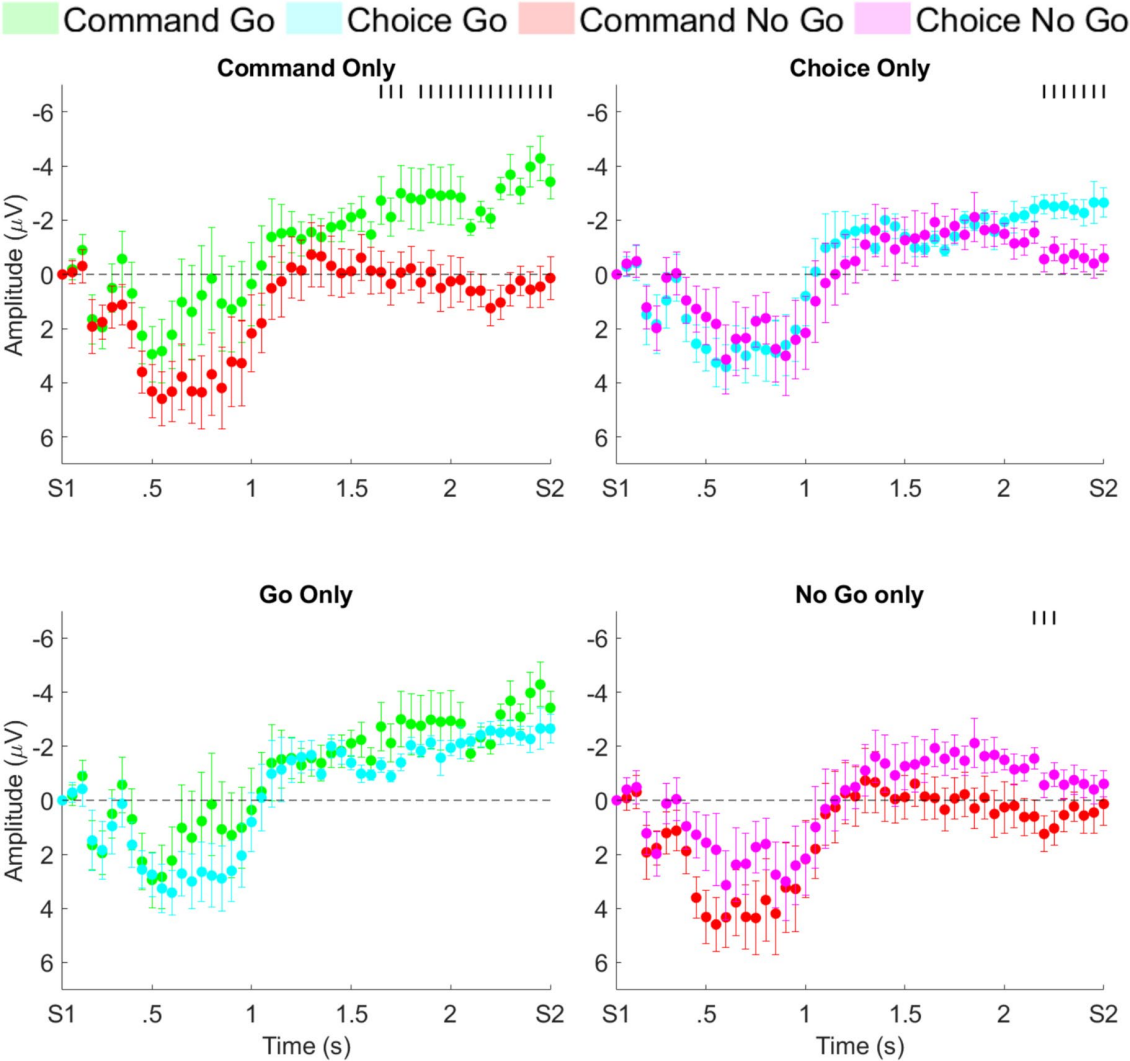
Bins, normalized to S1, averaging 50 ms between S1 and S2 comparing the command (top left), choice (top right), Go (bottom left), and No Go (bottom right) conditions for electrode Cz. For all plots, the black vertical lines at the top of each plot indicate statistical significance for that given bin

We then performed an analysis of additional time points of the CNV prior to the 50 ms window before S2 (Fig. 3). Specifically, we performed a post hoc comparison for 50 ms time blocks moving backward from S2. For the choice go and choice no-go tasks the differences extended for seven 50 ms bins, for a total of 350 ms (all p-values < 0.047), which was 2150 ms after S1. In the command conditions, there was a significant difference in three additional bins, occurring in the period 900 to 750 ms prior to S2 (all p-values < 0.0463), with the first difference then at about 1600 ms after S1. At the end of the CNV, the voltage topo-plots (Figure S4) showed the maximum voltage for both go conditions to move toward C3 while the maximum voltage for both no-go conditions was slightly anterior to Cz.

For the command go and choice go conditions, we observed no significant differences at the time of S2, nor extending back toward S1 (all comparisons, p > 0.05). For the command no-go and choice no-go, while there were no significant differences at the time of S2, there were three 50 ms bins that were significantly different from each other, occurring from 400 to 250 ms before S2 (all p-values < 0.0404).

The CNV for all 4 conditions is about the same in the first second and must reflect at least in part a similar response to S1 but does not reflect any decision being made.

### Alpha and beta power

**Fig. 4:**
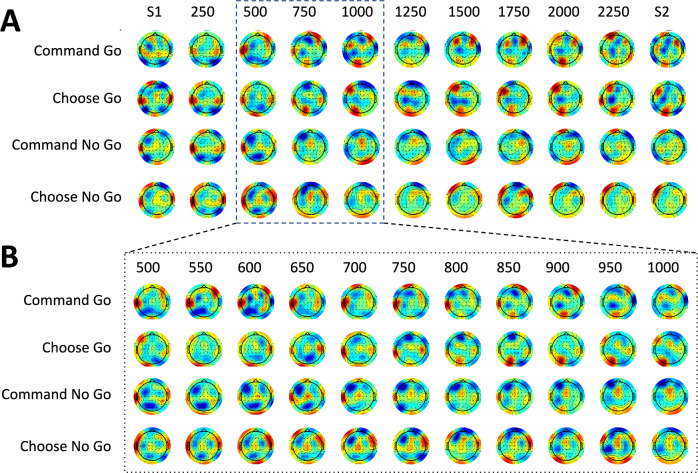
**A** Topo plot for beta activity between S1 and S2; **B** Topo plot for beta activity, zooming in on the timing between 500 and 1000 ms after S1

To further examine differences in brain activity during the command and choice tasks, we explored power in the full EEG and the alpha (7–12 Hz) and beta (18–24 Hz) ranges during the pre-S2 period over the full set of electrodes. From the full time–frequency spectrogram over Cz, we extracted mean activity in the alpha and beta bands and visualized mean power values in the period 50 ms before S2. We found higher alpha (t = − 2.830, p = 0.0163) and beta (t = − 2.891, p = 0.0161) power in the command no-go versus the command go conditions. However, we report no differences for the choice go and choice no-go conditions (both comparisons, p > 0.628). While we saw no major differences in the full EEG or the alpha band, the beta band appeared to be complex and informative (Fig. 4). Specifically, there was a difference in beta for choice-go and choice-no-go at 500 ms post S1 in the left DLPFC. As all the findings in beta are exploratory, we have summarized them in the supplementary material.

## Discussion

We used the CNV to look for signs of the decision making process for a free-choice decision to move (go) or not move (no-go) compared to that of commanded action or inaction. We found that free-choice and command CNVs were similar with the free-choice records lagging the command CNVs by short amounts of time. Thus, we see that when there is about 2500 ms to make a decision it takes about 1.5–2 s to do so. As an unexpected finding, we found that the CNV demarcation of a decision was “late” compared to much earlier apparent decisions in other parts of the brain. Thus, it appears that the CNV decision is actually more likely implementation of a movement decision that is made earlier.

Here we show that the CNV for command and choice tasks share similar responses in terms of amplitude at the time immediately before action, but differences in activity during the period leading up to this moment. The exploratory observations identify evidence that brain activity underlying beta modulation shows early brain activity that appears to play a significant role in the decision-making process well before the decision declares itself in the CNV. Our findings support the idea that initiating and canceling an action are closely related.

### Development of CNV

We found our a priori hypothesis to be true that there was a significant difference in the CNV amplitude in the 50 ms prior to S2 between the choice go and choice no-go conditions as well as the command conditions. In addition, we found that choice go and command go conditions had similar amplitude, as well as the choice no-go and command no-go conditions.

The post hoc inspection of the CNV waveforms over time seem to reveal patterns regarding the decision-making process. The relatively long interval between S1 and S2 allowed us to visualize the brain’s response to a commanded condition versus one in which a decision could be made about whether to move or to not move (Figs. 2, 3). Starting with S1, we first see the response to the visual cue in the first 1000 ms of the CNV waveform. At about 1000 ms we see that the command go conditions and the command no-go conditions begin to diverge, but this does not reach significance until 1600 ms (900 ms before movement). After divergence, the command no-go condition remains close to 0 µV and the command go condition moves toward a voltage of about − 5 µV.

During the choice tasks, we find that the CNV waveforms remain together and with a voltage in between the command go and no-go conditions, suggesting that a decision has not yet been made. The divergence between these conditions is in the same direction as the command conditions but happens much later, at approximately 2150 ms after S1 (350 ms before movement). Our findings of the CNV and alpha and beta ERD in the command conditions are similar to previous findings (Filipovic et al. 2001; Filevich et al. 2012), but there have not been previous studies with choice conditions.

Regarding the CNV, an apparent conclusion is that the decision for command conditions is made at 1600 ms after S1, and the decision in the choice conditions is not made until 2150 ms after S1. The exploratory beta topoplots, however, show the decision to be much earlier, and moreover, show how the brain goes from decision to action (Fig. 4). Hence, the CNV more likely indicates movement preparation than the decision process. We acknowledge that some participants might make their choice at S2 rather than at S1, as suggested by studies from Kuhn and others (Kuhn et al. 2009). This could contribute to the later CNV divergence observed between go and no-go trials for the free choice condition. To understand our results, it is necessary to review prior studies of go/no-go experiments with and without choice.

### Prior literature

There have been many prior studies using different variations of a go/no-go paradigm, mostly with fMRI. As the brain areas for movement are well known, the main interest in these studies is what happens in no-go situation. Results from all studies are consistent in showing areas that are more active with no-go than go indicating that inhibition is an active process. The specific areas vary to some extent among the studies, likely due to variations in the paradigms, but there is some consistency. The lateral part of the prefrontal cortex, either right (Dunovan et al. 2015; Aron et al. 2005) or left (Criaud et al. 2013; Brass et al. 2007; Kuhn et al. 2009) or bilateral (Watanabe et al. 2002) is most commonly identified. This region is described as inferior frontal gyrus (IFG), mid-frontal gyrus (MFG), left dorsal frontomedian cortex (dFMC), or dorsolateral prefrontal cortex (DLPFC). The pre-supplementary motor area (preSMA) is frequently noted (Dunovan et al. 2015; Simmonds et al. 2008), and sometimes premotor cortex (PMC) (Watanabe et al. 2002). One study noted directed connectivity from the left dorsal MFG to the preSMA (Kuhn et al. 2009). The inferior parietal lobule (IPL) is also frequently noted, left (Criaud et al. 2013; Watanabe et al. 2002; Brass et al. 2007) more often than right (Kuhn et al. 2009). These studies have identified important structures, but do not reveal their time course. Other than the two studies noted above that looked at the CNV with command instructions, there are no EEG studies. Takeyama et al. (Takeyama et al. 2022) studied no-go event-related potentials (ERPs) with intracranial EEG (ECoG) and found activity in the left posterior MFG and in the preSMA, SMA, and PMC regions. They then stimulated these areas and found inhibitory effects.

Recent studies have further explored the impact of varying Go/No-Go ratios on inhibition-related brain activity. For example, an event-related potential (ERP) study found that different ratios of Go and No-Go trials significantly influence behavioral performance and brain activity related to response inhibition. As the proportion of Go trials decreased, behavioral performance in Go trials improved, and error rates in No-Go trials decreased, highlighting the modulatory effect of trial ratios on inhibition-related neural processes (Zhang et al. 2024).

Furthermore, recent research has highlighted the differences between Go/No-Go and Stop-Signal tasks in terms of their neural and cognitive mechanisms. While both tasks measure inhibitory control, Go/No-Go tasks are more reflective of automatic inhibition, whereas Stop-Signal tasks are indicative of controlled inhibition. Neuroimaging studies have shown that these tasks engage overlapping but distinct neural circuits (Sebastian et al. 2013). Additionally, cognitive studies have shown that external factors, such as negative stimuli, can differentially modulate performance in these tasks, further supporting the idea that they rely on different inhibitory processes (Littman and Takács 2017).

There are fewer fMRI studies dealing with free choice in a go/no-go paradigm. Si et al. (2021) reported a meta-analysis that revealed bilateral anterior cingulate and preSMA, bilateral prefrontal cortex (PFC), bilateral IPL, and right PMC. Kuhn et al. (2009) found specific activation in anterior cingulate. EEG was reported in such a free choice experiment also (Leocani et al. 2001), but data were not analyzed to identify the pattern of relevant regions. In many other types of free choice paradigms, the mesial motor areas are typically involved. The conclusion is that there are many studies, but most deal with neuroimaging, and the time course of events is difficult to discern with that modality.

### Limitations

A limitation of our study is the small sample size, which may reduce statistical power and increase the risk of Type II errors. This could lead to an underestimation of the true effects or failure to detect smaller but meaningful differences. However, this sample size is consistent with other similar studies in the field (e.g., do Nascimento et al. 2006; Brass and Haggard 2007; Shakeel et al. 2015). Future studies with larger cohorts are needed to confirm and extend these findings.

Due to the exploratory nature of our analysis and the limited number of trials, we were unable to perform a statistical analysis of the ERD topoplots. The complexity of the variables—frequency, time, channel, and condition—further complicated the analysis. As a result, our findings regarding the ERD are qualitative in nature. Future studies with a more focused scope and increased trial numbers may allow for a more detailed statistical exploration of these phenomena.

This study was initially designed to focus on the CNV, with the analysis of full brain activity included as an exploratory component. We had no prior hypothesis about the specific value of the beta activity, which unexpectedly emerged as particularly informative. Further experiments of this type, with better quantification and variations in movement tasks, would be valuable. Collecting sufficient data for each participant would also enable the examination of individual differences, which could provide additional insights.

Additionally, we were unable to perform source analysis and instead relied on prior literature to infer the brain areas most active in tasks of this type. Conducting the same experiments with fMRI in the same participants would help to refine our understanding of the anatomical locations involved in these processes.

## Supplementary Information

Below is the link to the electronic supplementary material.

Supplementary file1 (DOCX 29 KB)

Supplementary file2 (PDF 2062 KB)

## Data Availability

Data will be made available upon reasonable request.
