# Supplementary material for: Patterns of brain activity in choice or instructed go and no-go tasks
Source: Exp Brain Res. 2025 Feb 21;243(3):73. doi: 10.1007/s00221-025-07027-6 (PMC11845411; doi:10.1007/s00221-025-07027-6)
Supplement: Supplementary file 1 — Supplementary file1 (DOCX 29 KB) [file 221_2025_7027_MOESM1_ESM.docx]

**Supplementary material:**

**Results**

Commentary on Alpha and Beta Power

See Figure 4

As an exploratory study, we also visualized alpha and beta activity during the full time period between S1 and S2 over the whole scalp. While alpha activity did not reveal any notable patterns to differentiate the task conditions, we found that beta activity appeared unique for the different conditions starting approximately 500 ms after S1. At 250 ms after S1, there did not appear to be much difference between the 4 conditions, and the bilateral occipital event related desynchronization (ERD) was presumably due to the S1 visual stimulus. Beginning at approximately 500 ms there was a clear difference between the command go and command no-go, indicating the initiation of the different plans. The no-go topography showed a brief beta ERD at F3 and then a strong ERD at AF7/F7 that lasted until about 1250 ms. From about 600 ms to 1000 ms, there was also an event related synchronization (ERS) beginning around Cz and moving anterior to Fz before fading out. From about 1500 ms, there was no distinctive beta activity. The go topography showed ERD at F3 beginning 50 ms later than the no-go, but then the ERD remained at that location and enhanced for about 100 ms. At about 700 ms there was an ERD at C3 that lasted until the movement was generated at S2. During the same period, there was an ERD at AFz/FPz that migrated slightly posterior to Fz/AFz at the time of S2. Beginning at about 1000 ms, at AF7/F7, where there was a strong ERD for the no-go condition, there was an event related synchronization (ERS) for the go condition which lasted almost to the time of S2.

The choose no-go condition demonstrated beta topography that was similar to that of the command no-go condition but started weaker and often seemed to develop approximately 50 to 100 ms behind the choice no-go condition. From about 900 ms to 1750 ms, there was an intermittent ERD at C5, which was perhaps stronger in the choice no-go condition. Generally, however, from about 750 ms, there were no major differences between the command no-go and choice no-go conditions.

The choice go condition did not have any strong activation at F3 but did show a trace of that activity which started around 600 ms, 50 ms later than the command go, and lasted only a short time. However, at about 700 ms, the choice go condition matched the command go condition with ERD at AFz/FPz that lasted, although weaker, until S2. Additionally, there was ERD at C3, starting at about 750 ms, which was weak at first but then became just as strong as that seen in the command go condition by 1250 ms. There was also the ERS at AF7/F7 beginning at about 1000 ms, carrying through to S2, possibly stronger than that of the command go condition. From about 1000 ms to S2, there were no major differences between the command go and choice go conditions.

The difference between the choice go and choice no-go conditions showed a weak difference at 550 ms which became clear by 600 ms. The distinguishing topographic finding was present in the choice no-go condition, where the ERD at F3 moved to AF7/F7. Moreover, by 650-700 ms, the ERS at Cz in the choice no-go condition was also different. The choice go topography became distinctive at 700 ms with the ERD at AFz/FPz. Although the timing was slightly different, the ERD at F3 was similar for the two choice conditions. The first major distinction was the ERD at AF7/F7 at 700 ms in the choice no-go condition.

**Discussion**

Commentary on Alpha and Beta Power

Beta changes and the decision-making process

The exploratory beta activity looks to be highly informative, indicating decision times much earlier than what is decoded from the CNV, and appears to show the development of the go and no-go tasks following the decision. Our data are scalp electrode sites and what is needed is sources. For this exploratory study, we will assume that the activity arises from the cortex directly underneath the electrode. Table 1 shows the informative electrode sites in our study and Figure S5 shows the related cortical areas as determined by an anatomical investigation of the 10-10 system (Koessler et al. 2009).

Table 1: Likely brain regions that are sources of activity at electrode sites relevant in study data. Information in the first three columns comes from Cohen 2014 and Koessler et al 2009.

| **Electrode** | **Brain Area Location** | **Brodmann Area** | **Likely Brain Region** |
| --- | --- | --- | --- |
| AF7 | Midfrontal gyrus | 10 | Dorsolateral prefrontal cortex (DLPFC) |
| AFz | Bilateral medial frontal | 9 | Pre-supplementary motor cortex (preSMA) |
| Cz | Medial precentral gyrus | 4 | Supplementary motor cortex (SMA) |
| C3 | Lateral postcentral gyrus | 1, 2,3 | Sensorimotor cortex (M1/S1) |
| C5 | Lateral postcentral gyrus | 1,2,3 | Sensorimotor cortex (M1/S1) |
| Fz | Bilateral medial frontal | 6 | Pre-Supplementary motor cortex/Supplementary motor cortex (preSMA/SMA) |
| F3 | Midfrontal gyrus | 8 | Dorsolateral prefrontal cortex (DLPFC) |
| F7 | Inferior frontal gyrus | 45 | Dorsolateral prefrontal cortex (DLPFC) |
| FPz | Bilateral medial frontal | 10 | Anterior cingulate cortex (ACC) |

Interpreting our data considering previous literature, it appears that the first manifestation of the brain’s differentiated response to S1 is for the command conditions in the left DLPFC. This is an ERD that appears slightly earlier for the command no-go (500 ms) than for the command go (550 ms). For the command no-go, this is quickly followed by ERS activity in preSMA/SMA at 600 ms, which lasts until about 1000 ms before fading off. Such rapid information transfer for inhibition could be mediated by the directed connectivity noted earlier (Kuhn et al. 2009). ERD activity in the left DLPFC moved to a more anterior-ventral region (AF7/F7) and lasted until 1250 ms. For the command go condition, the left DLPFC ERD lasts about 100 ms, then fades, but at about 1000 ms, activity reappears in the AF7/F7 area, but as an ERS, opposite to what is seen with no-go, and this ERS lasts almost to the time of S2. Following the left DLPFC activity, at about 700 ms ERD appears in the left sensorimotor area and remains there until the movement occurs. Additionally at the same time, an ERD begins in the preSMA and that moves toward the SMA at the time of S2. The CNV for the two conditions seems to represent mainly the activity in the preSMA and SMA, perhaps with some influence as well from the contralateral sensorimotor area. However, the brain activity during the go and no-go tasks is clearly better illuminated by the beta activity.

The pattern of activity of the two choice conditions is best described as starting slightly later than the command conditions, being slightly weaker in activation at first, but eventually matching their corresponding command conditions.

The decision for go or no-go appears to arise in the left DLPFC. There is no ambiguity in the command conditions and the activity arises rapidly. The plan is maintained in another place in the DLPFC, more anterior and ventral, and represented by opposite beta power changes for go and no-go. Additionally for no-go, the plan is relayed to the preSMA/SMA where it is also maintained until S2. Additionally, for go, the plan is relayed to the primary sensorimotor cortex where it is maintained until its action is consummated at S2. The second addition for go is the activation of the preSMA/SMA, more anterior to that of the no-go activation, and which also remains until S2. The production of the movement appears to be cooperative action of the sensorimotor cortex and preSMA/SMA.

The decision for choice go or no-go arises slightly later and weaker in the left DLPFC, but eventually builds to the same level of activity as the command conditions. Our data are averages, and the “sluggishness” may be real or reflect a combination of decisions at variable time after S1. In any event, there is no “decision area” that could be defined prior to the activity in the left DLPFC in precisely the same place as for the command conditions. Thus, it appears that in a brain primed simply for making or not making a movement, the same area analyzes S1 and on the basis of its pre-set state, yields the decision. We would also conclude that the CNV does not indicate the decision process itself, but the differential preparation of the upcoming action.
