# Supplementary figures and images for: Patterns of brain activity in choice or instructed go and no-go tasks
Source: Exp Brain Res. 2025 Feb 21;243(3):73. doi: 10.1007/s00221-025-07027-6 (PMC11845411; doi:10.1007/s00221-025-07027-6)

**A**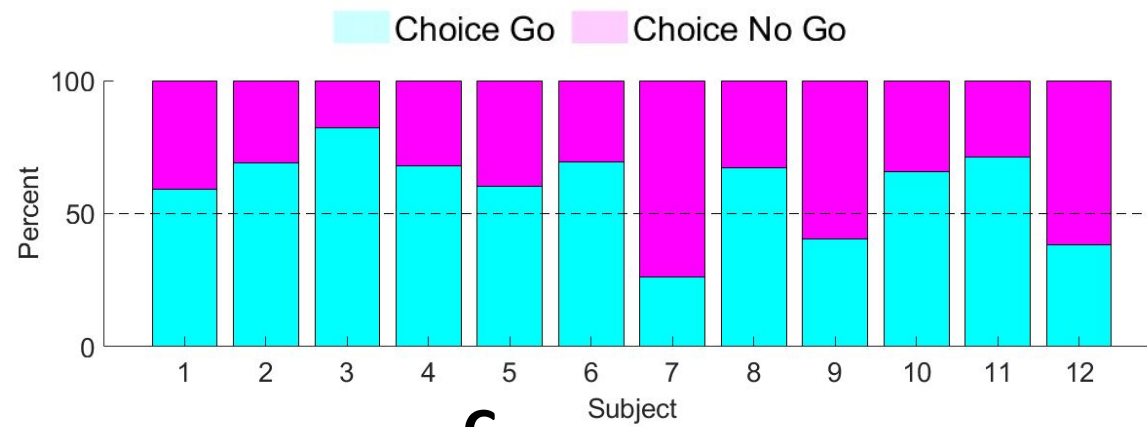**B**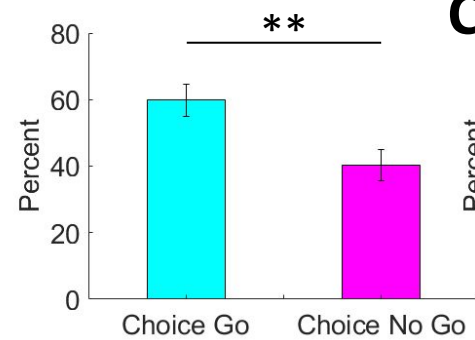**C**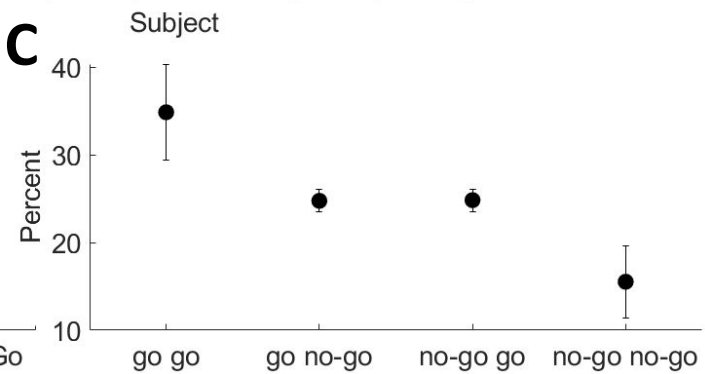

**A**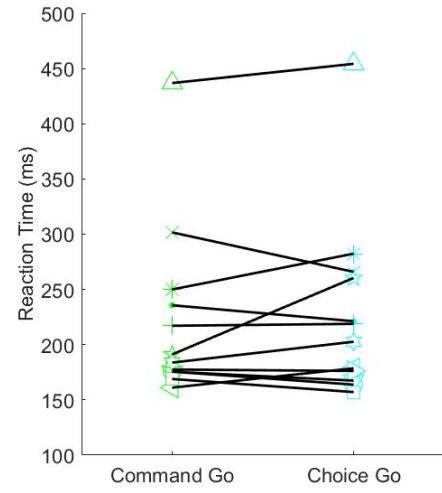**B**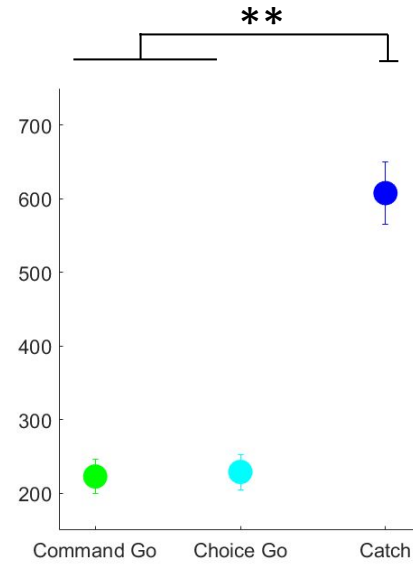

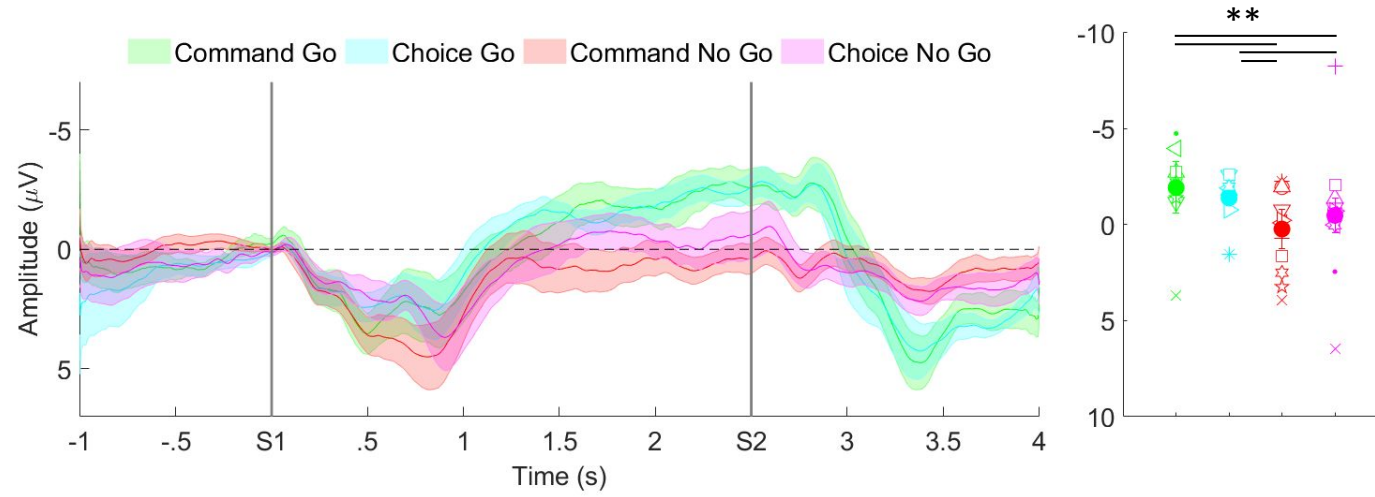

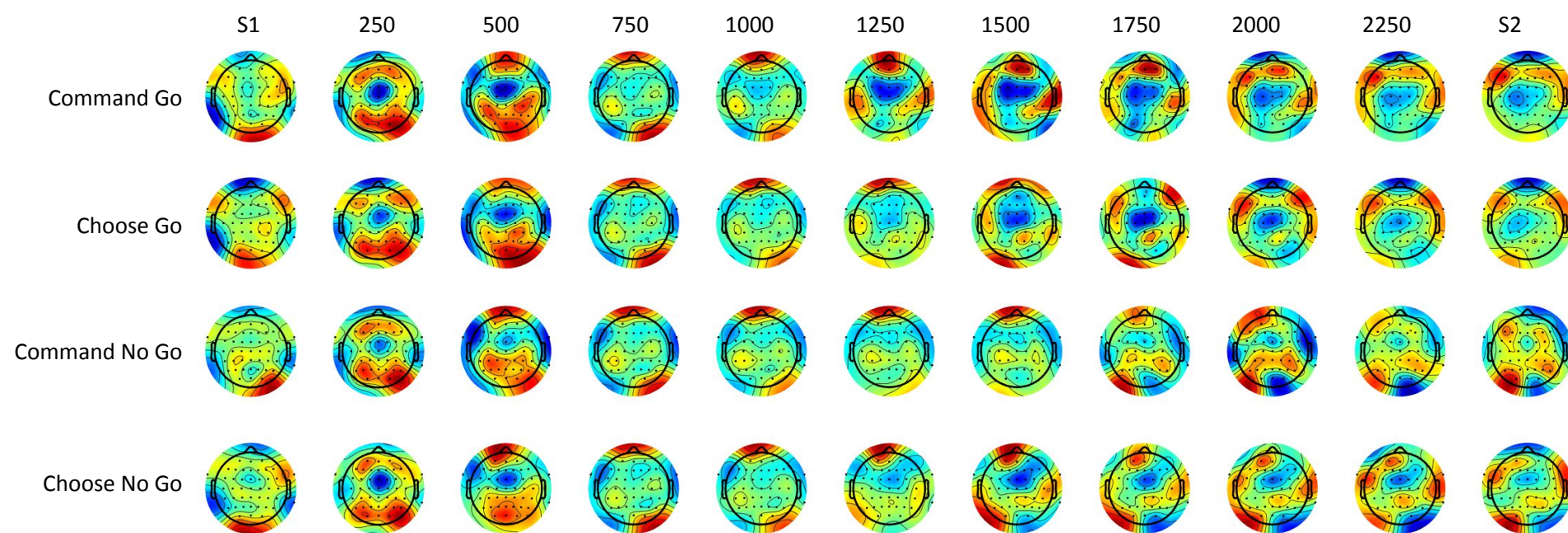

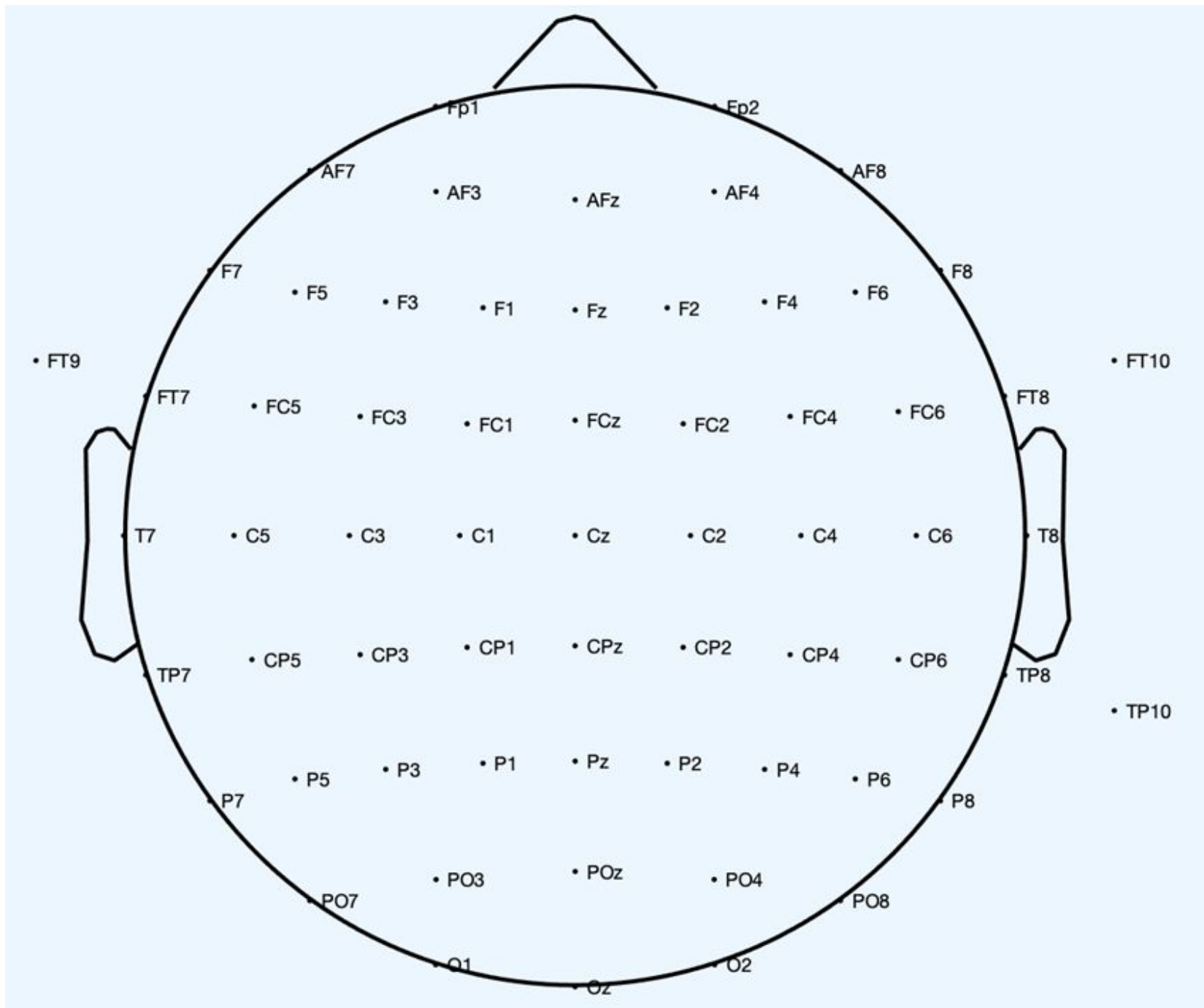

Supplement: Supplementary file 2 — Supplementary file2 (PDF 2062 KB) [file 221_2025_7027_MOESM2_ESM.pdf]
